# Formyl peptide derived lipopeptides disclose differences between the receptors in mouse and men and call the pepducin concept in question

**DOI:** 10.1371/journal.pone.0185132

**Published:** 2017-09-21

**Authors:** Malene Winther, André Holdfeldt, Martina Sundqvist, Zahra Rajabkhani, Michael Gabl, Johan Bylund, Claes Dahlgren, Huamei Forsman

**Affiliations:** 1 Department of Rheumatology and Inflammation Research, Institute of Medicine, Sahlgrenska Academy, University of Gothenburg, Gothenburg, Sweden; 2 Department of Oral Microbiology and Immunology, Institute of Odontology, Sahlgrenska Academy, University of Gothenburg, Gothenburg, Sweden; University of North Dakota, UNITED STATES

## Abstract

A pepducin is a lipopeptide containing a peptide sequence that is identical to one of the intracellular domains of the G-protein coupled receptor (GPCR) assumed to be the target. Neutrophils express two closely related formyl peptide receptors belonging to the family of GPCRs; FPR1 and FPR2 in human and their respective orthologue Fpr1 and Fpr2 in mouse. By applying the pepducin concept, we have earlier identified FPR2 activating pepducins generated from the third intracellular loop of FPR2. The third intracellular loop of FPR2 differs in two amino acids from that of FPR1, seven from Fpr2 and three from Fpr1. Despite this, we found that pepducins generated from FPR1, FPR2, Fpr1 and Fpr2 all targeted FPR2 in human neutrophils and Fpr2 in mouse, but with different modulating outcomes. Whereas the FPR1/Fpr1 derived pepducins inhibited the FPR2 function in human neutrophils, they activated Fpr2 in mouse. The FPR2 derived pepducin activated FPR2/Fpr2, whereas the pepducin generated from Fpr2 inhibited both FPR2 and Fpr2. In summary, our data demonstrate that pepducins generated from the third intracellular loop of human FPR1/2 and mouse Fpr1/2, all targeted FPR2 in human and Fpr2 in mouse. With respect to the modulating outcomes, pepducin inhibitors identified for FPR2 are in fact activators for Fpr2 in mouse neutrophils. Our data thus questions the validity of pepducin concept regarding their receptor selectivity but supports the notion that FPR2/Fpr2 may recognize a lipopeptide molecular pattern, and highlight the differences in ligand recognition profile between FPR2 and its mouse orthologue Fpr2.

## Introduction

The members of the formyl peptide receptor (FPR) family expressed by neutrophil phagocytes belong to the large group of G-protein coupled receptors (GPCRs) and play key roles in proper recruitment and activation of neutrophils at sites of infection/inflammation [1–3]. Neutrophil can be activated by many stimuli, including FPR agonists, and generally such activation leads to a release of reactive oxygen species and granule constitutes. This is beneficial if production/release is properly controlled but is associated with an increased risk for damage to the surrounding cells/organs/tissues if the process gets out of control [4–7]. A delicate balance of FPR-mediated initiation and resolution of inflammation is thus required for successful clearance of microbes and tissue debris, while at the same time limiting inflammation associated tissue damaging.

When looking at the FPRs in different species it is clear that the receptor family has a complex evolutionary history, as illustrated by the fact that the number of genes in the family vary markedly in mouse and man [8]. Human phagocytes express two (neutrophils) or three (monocytes) FPRs, whereas the mouse Fpr family comprises at least eight members among which Fpr1 and Fpr2 are expressed by mouse neutrophils and are the suggested orthologous of the human FPR1 and FPR2, respectively [9–11]. As different mouse models constitute important tools for translational studies aiming to understand the patho-physiological roles of FPRs, a better characterization of mouse Fprs in the form of ligand recognition by agonists/antagonists/modulators known to regulate activities of the human receptors is thus needed. We have started this work and previously identified two formylated peptide agonists generated by *Staphylococcus aureus* bacteria that potently and selectively activate Fpr1 (fMIFL) and Fpr2 (PSMα2), respectively [12]. We also recently showed that some of the most potent and selective antagonists for the human FPRs lack effects on their mouse counterparts, cyclosporin H and PBP_10_ being prominent examples [12]. We have also identified lipidated peptidomimetics and a set of formylated MHC class Ib binding peptides as novel agonists for both human and mouse receptors, but there were no direct correlations between the activities induced by these agonists in human and mouse neutrophils [13, 14]. This clearly implies that when comparing mice and men there are some similarities between the receptor orthologous in the two species, but there are also important differences in the ligand recognition profiles.

When searching for new mechanistic concepts for allosteric modulation of GPCRs in the early 21^th^ century, a unique class of lipopeptide ligands (pepducins) was introduced. The proposed concept for interaction suggested that pepducins modulate receptor signaling through a direct interaction between the peptide part of the pepducins and intracellular signaling active parts of the targeted receptors [15–17]. Pepducins contain a short peptide sequence N-terminally linked to a palmitic acid and in order to achieve receptor specificity, the amino acid sequence of the pepducin should be identical to one of the intracellular domains (one of the loops or the cytoplasmic tail) of the receptor to be targeted. Compared to the binding characteristic of conventional ligands that mediate their function through a direct interaction with receptor parts exposed on or close to the cell surface, the suggested mode of action for pepducins is unique. To achieve this distinctive mode of action, a pepducin needs to transverse the plasma membrane and interact with the receptor from the cytosolic side, and the receptor specificity of an allosterically modulating pepducin is proposed to rely on the sequence identity/similarity between the pepducin and the corresponding intracellular domain of the targeted receptor [16–18]. The outcome of an interaction between the two identical peptide sequences present in the modulator and the receptor, respectively, will be either an activation or an inhibition of receptor function [15]. Over the last 15 years, the pepducin concept has been shown to be valid for a number of receptors, including FPR2, for which both activating and inhibiting pepducins have been described [19–22]. It should be noticed, however, that the basic interacting characteristics when it comes to activation/inhibition induced by FPR pepducins are not always in agreement with the proposed pepducin concept [2]. This is illustrated by the fact that pepducins derived from FPR1, CXCR4 and the ATP receptor (P2Y_2_R) all have in common that they modulate FPR2 function [23–25]. The effects of FPR2 activating pepducins are also inhibited by conventional FPR2 antagonists, results not in agreement with the pepducin concept [20]. Even though the precise mechanism of action for FPR2 modulating pepducins has not yet been elucidated, it is clear that pepducins are unique tools to modulate FPR2 functions.

In this study, we applied the pepducin approach in an attempt to gain more insights into similarities and differences in the ligand recognition profiles of the human neutrophil FPRs and their mouse orthologous. We have determined the effects of two earlier described human FPR pepducins generated from the third intracellular loop of FPR1 (F1Pal_16_) and FPR2 (F2Pal_16_) on mouse neutrophils [20, 23]. The modulating effects of the corresponding pepducins derived from mouse Fpr1 (mF1Pal_16_) and Fpr2 (mF2Pal_16_) on mouse and human neutrophils were also investigated. We show that the FPR2 derived pepducin activated both FPR2 and Fpr2, whereas the corresponding Fpr2 pepducin potently inhibited FPR2/Fpr2. The FPR1- as well as the Fpr1-derived pepducins interacted with FPR2 and Fpr2, but the outcome of this interaction differed as they activated Fpr2 but inhibited FPR2 function. Taken together, we describe pepducins that inhibit FPR2 activity in one species activate the receptor orthologue in another species, and that all the modulating pepducins studied selectively targeted FPR2/Fpr2 irrespectively from which the receptor the peptide sequence was derived. Although our results disagree with the proposed molecular mechanism for how pepducins achieve their receptor specificity, this group of FPR2 selective ligands clearly serve as excellent tools for further studies of FPR2/Fpr2 functions.

## Materials and methods

### Chemicals

Percoll was obtained from Amersham Pharmacia (Uppsala, Sweden). Dextran and Ficoll-Paque were obtained from GE-Healthcare Bio-Science (Uppsala, Sweden). The peptides were synthesized and purified by high-pressure liquid chromatography (HPLC) by Alta Bioscience (University of Birmingham, United Kingdom). Cyclosporin H (CysH) was kindly provided by Novartis Pharma (Basel, Switzerland) and the peptidomimetic activator F2M2 and inhibitor were kindly provided by Henrik Franzyk (Copenhagen, Denmark). Boc-FLFLF (Boc2) and isoluminol were purchased from Sigma Chemical Co. (St. Louis, MO). The phenol-soluble modulin (PSMα2, fMGIIAGIIKFIKGLIEKFTGK) was obtained in its N-formylated form from EMC (Tübingen, Germany). Recombinant murine tumor necrosis factor alpha (TNF-α) was from R&D Systems Europe Ltd. (Abingdon, Oxon, United Kingdom). Horseradish peroxidase (HRP) was from Boehringer Mannheim (Mannheim, Germany). Pepducins and the FPR2 inhibitor PBP_10_ were from Caslo Laboratory (Lyngby, Denmark). The pepducins were synthesized by Fmoc solid-phase peptide synthesis and the fatty acid were N-terminally linked on the resin as the last step before deprotection of side chains, followed by HPLC purification on a C18 column and further verification by MALTI-TOF Mass Spectrometry. All peptides and pepducins were dissolved in dimethyl sulfoxide to a concentration of 10 mM and stored at -80°C until use. Further dilutions were made in Krebs-Ringer phosphate buffer that was supplemented with glucose (10 mM), Ca^2+^ (1 mM), and Mg^2+^ (1.5 mM) (KRG; pH 7.3).

### Animals and ethics statement

C57BL/6 wild-type mice were purchased from Charles River Laboratories and Fpr2^−/−^ mice generated as described previously [26], were generously provided by Dr Ji Ming Wang, Frederick National Laboratory for Cancer Research, USA. Mice were kept under standard temperature and light conditions and fed laboratory chow and water *ad libitum*, at the Department of Rheumatology and Inflammation Research, University of Gothenburg. Sex matched WT and Fpr2^-/-^ animals (age 8–10 weeks) were used to isolated bone marrow derived neutrophils. The studies performed were approved by the Ethical Committee for Animal Experimentation, Göteborg, Sweden.

### Isolation of human neutrophils from peripheral blood and ethics statement

Human peripheral blood neutrophils were isolated from buffy coats from healthy blood donors using dextran sedimentation and Ficoll-Paque gradient centrifugation as described [27]. The remaining erythrocytes were disrupted by hypotonic lysis and the neutrophils were washed twice, suspended in KRG and, stored on melting ice until use. This isolation process permits cells to be purified with minimal granule mobilization. The buffy coats were obtained from the blood bank at Sahlgrenska University Hospital. Ethics approval was not needed since the buffy coats were provided anonymously and could not be traced back to a specific individual. This is in line with Swedish legislation section code 4§ 3p SFS 2003:460 (Lag om etikprövning av forskning som avser människor).

### Preparation of polymorphonuclear leukocytes from bone marrow

Mice (8–12 weeks of age) were killed by cervical dislocation, the femurs and tibias were removed and freed of soft tissue attachments, and the extreme distal tip of each extremity was cut off. KRG without Ca^2+^ and Mg^2+^ (KRG-) was forced through the bone by using a 1-mL syringe with a 27-gauge needle. After dispersing cell clumps and removing the debris, the bone marrow granulocytes were isolated according to an earlier described procedure [28] with some modifications. Briefly, a cell suspension in 2 mL of KRG- was laid on top of a three-layer Percoll gradient (1.095, 1.085, and 1.070 g/mL). The density of each Percoll solution was verified using density marker beads. After centrifugation at 500 × g for 30 min at 4°C in a swinging bucket rotor, the lowest band (1.085/1.095 g/mL interface) was collected as the neutrophil fraction. After washing with KRG-, remaining red blood cells were eliminated by hypotonic lysis. After a final wash with KRG-, the cells were resuspended in KRG. The cell number and population of bone marrow cells were determined by flow cytometry based on forward scatter (size) and side scatter (density) localization.

### Measurement of NADPH-oxidase activity

NADPH-oxidase activity was determined using an isoluminol and HRP-enhanced chemiluminescence (CL) system that allow for the determination of superoxide production [29, 30]. The CL activity was measured in a six-channel Biolumat LB 9505 apparatus (Berthold Co., Wildbad, Germany), using disposable 4-mL polypropylene tubes with 1 mL reaction mixture. The tubes contained 4 U HRP and 10 μg/mL isoluminol together with the cells that were equilibrated in the Biolumat for 5 min at 37°C, after which the stimulus solution (100 μL) was added. The light emission was recorded continuously over time and presented as superoxide production (in arbitrary light units, cpm x10^-6^) as a function of time (min). The activity before stimulus addition was recorded as basal line and this activity was not changed in vehicle control samples. Since the FPR/Fpr-mediated superoxide production with respect to the onset and decline are very similar, the peak activities were used to compare the responses induced.

Naïve bone marrow cells produce very low levels of superoxide upon stimulation and to increase this production the cells were routinely primed for one hour at RT followed by TNF-α (10 ng/mL, final concentration) priming for a period of 20 min at 37°C [10]. The cells were then kept on ice until further analysis, as described above. The reason for choosing the chemiluminescence based NADPH-oxidase assay system was that very few cells are required [31], allowing us to monitor and quantify receptor-mediated events with the limited number of mouse bone marrow neutrophils available.

### Data analysis and statistics

Data analysis was performed using Graph Pad Prism 7.0. The EC_50_/IC_50_ values were determined from experiments in which the activities were normalized to the response induced by maximal response or induced by agonist in the absence of inhibitor. The statistical analysis was performed on raw data using Student’s *t*-test when two groups were compared or one-way ANOVA with Dunnett’s multiple comparisons test in comparison to the control response when three groups were compared. * *p* ≤ 0.05 was considered significant.

## Results

### The F2Pal_16_ pepducin generated from the third intracellular loop of FPR2 positively modulates FPR2 and the mouse orthologue Fpr2

**Fig 1:**
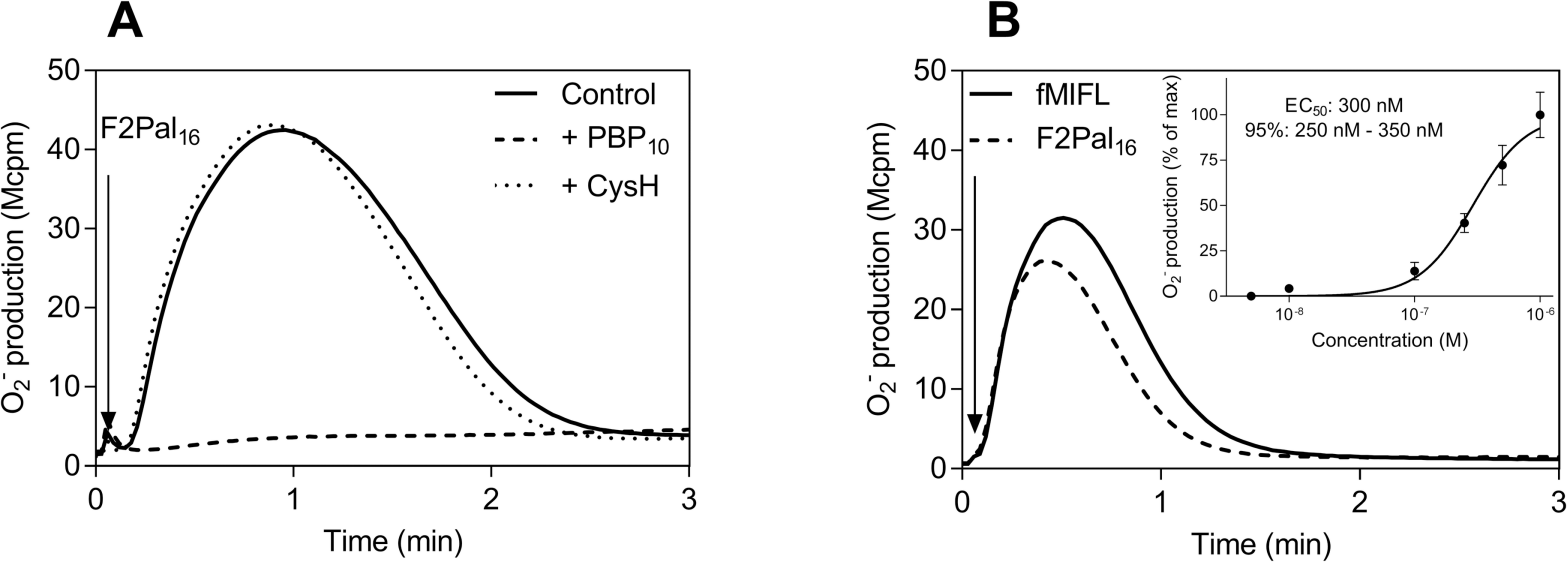
The F2Pal_16_ pepducin generated from FPR2 activates both human and mouse neutrophils to produce superoxide. **A)** Human neutrophils (10^5^ cells) were activated by the pepducin F2Pal_16_ (1 μM, indicated by the arrow) in the presence of the FPR2 inhibitor PBP_10_ (1μM, dashed line) or the FPR1 antagonist CysH (1 μM, dotted line) or left untreated (solid line). The antagonists were pre-incubated with human neutrophils for five minutes in the presence of HRP and isoluminol before F2Pal_16_ addition and the release of superoxide anions was continuously measured. The figure shows one representative experiment out of five independent experiments performed with individual buffy coats. **B)** Mouse neutrophils (5 x 10^4^ cells) were stimulated with F2Pal_16_ (1 μM, solid line) and the release of superoxide anions was continuously measured. The response induced by FPR1 agonist fMIFL (10 nM, dashed line) is shown for comparison. **Inset:** Dose-dependent activation of WT mouse neutrophils by F2Pal_16_. The peak values obtained from different concentrations of F2Pal_16_ were normalized to the max response and Curve fitting was performed by non-linear regression using the sigmoidal dose-response equation (variable-slope, HillSlope 2). EC_50_ value and 95% confidence interval (CI) are calculated from three independent experiments with individual mouse (mean ± SD).

We have previously shown that the FPR2 derived pepducin (F2Pal_16_, Pal-KIHKKGMIKSSRPLRV), with a peptide sequence identical to the entire third intracellular loop of FPR2 (spanning from amino acid K_227_ to V_242_), activates human neutrophils (Fig 1A, [20]). Despite the large sequence similarity between FPR2 and the closely related FPR1 in the third intracellular loop (differing in two amino acids) from which the pepducin was generated (Table 1), the F2Pal_16_ pepducin selectively targeted FPR2 as the activity was completely blocked by the established FPR2 inhibitor PBP_10_ but not by the FPR1 inhibitor cyclosporine H (Fig 1A).

**Table 1:** List of the four formyl peptide receptors and their corresponding pepducins investigated in this study.

Receptor	FPR1	FPR2	Fpr1	Fpr2	3^rd^ intracellular loop	Pepdcin
**FPR1**	100	69	72	64	KIHKQGLIKSSRPLRV	F1Pal_16_
**FPR2**	69	100	64	76	KIHKKGMIKSSRPLRV	F2Pal_16_
**Fpr1**	72	64	100	63	KIHRQGLIKSSRPLRV	mF1Pal_16_
**Fpr2**	64	76	63	100	KINRRNLVNSSRPLRV	mF2Pal_16_

The upper panel show sequence identity at the amino acid level between the two human neutrophil FPRs and their mouse orthologues. The peptide sequences in the third intracellular loop of FPRs/Fprs are listed in the lower panel and that is the region from which FPR/Fpr pepducins are designed.

The effect of the F2Pal_16_ pepducin was also determined in neutrophils isolated from wild type mice using superoxide release as readout system. Addition of F2Pal_16_ to mouse neutrophils triggered superoxide release (Fig 1B), suggesting a positive modulating effect of F2Pal_16._ The activation kinetics was very similar to that induced by earlier described Fpr agonists fMIFL (Fpr1) and PSMα2 (Fpr2) derived from *S*.*aureus* (the fMIFL response is shown in Fig 1B). The F2Pal_16_ pepducin induced response reached a maximum in mouse neutrophils at a concentration of 1 μM with an EC_50_ value ~ 300 nM (Fig 1B inset). According to the pepducin concept, there should be large amino acid sequence similarities between a modulating pepducin and the intracellular domains of the receptor that is targeted [16]. One would thus assume that the mouse receptor targeted (if any) should be Fpr1 (differs in 3 amino acids from that of F2Pal_16_ in the third intracellular loop) rather than Fpr2 (Table 1).

**Fig 2:**
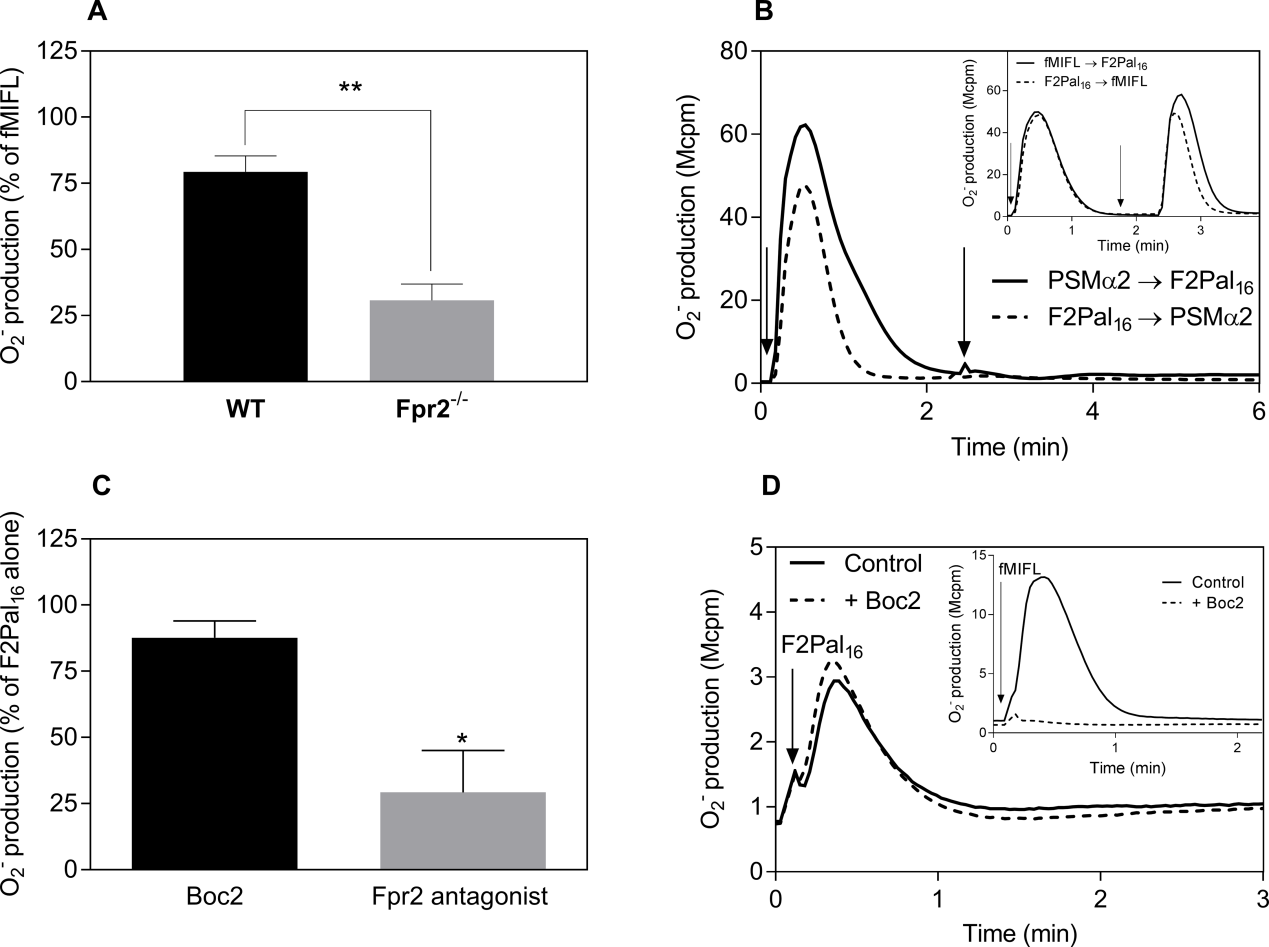
The F2Pal_16_ pepducin activates Fpr2 in mouse neutrophils. **A)** The levels of F2Pal_16_ (1 μM) triggered superoxide release were compared between WT neutrophils (black bar) and cells deficient in Fpr2 (Fpr2^-/-^, grey bar). The data are presented as % of the peak fMIFL (10 nM) response (a reference response that is comparable in WT and Fpr2 deficient neutrophils). **B)** WT mouse neutrophils (5 x 10^4^ cells) were first activated by addition of the peptide PSMα2 (50 nM, solid line indicated by the first arrow) or pepducin F2Pal_16_ (1 μM, dashed line indicated by the first arrow) and when the responses had declined, these cells received a second stimulation with F2Pal_16_ (1 μM, solid line, indicated by the second arrow) or PSMα2 (50 nM, dashed line, indicated by the second arrow). The release of superoxide anions was continuously measured. The figure shows one representative experiment out of four independent experiments from individual mouse. **Inset:** Lack of cross-desensitization between fMIFL and F2Pal_16_ in mouse neutrophils. Cells first received fMIFL (10 nM, solid line, indicated by the first arrow) or F2Pal_16_ (1 μM, dashed line, indicated by the first arrow) and when the response had declined, a second stimulation (second arrow) was induced by F2Pal_16_ (1 μM, solid line) or fMIFL (10 nM, dashed line). **C)** Effect of Fpr1 selective inhibitor Boc2 (2 μM, black bar, pre-incubated for 5 min) and Fpr2 selective peptidomimetic inhibitor (1 μM, grey bar, preincubated for 5 min) on the F2Pal_16_ response. Data are from five different experiments (mean ± SD). One-way ANOVA with Dunnett’s multiple comparisons test in comparison to the F2Pal_16_ control response was used for statistics, * *p ≤* 0.05. **D)** Fpr2^-/-^ neutrophils (5 x 10^4^ cells) were stimulated with F2Pal_16_ (500 nM) in the absence (solid line) or presence of the Fpr1 inhibitor Boc2 (2 μM, pre-incubated for 5 min). **Inset:** The fMIFL response (10 nM) in Fpr2^-/-^ neutrophils in the absence (solid line) or presence of Boc2 (2 μM, dashed line, pre-incubated for 5 min). The figure shows one representative experiment out of four independent experiments from individual mouse.

The tools used to elucidate the receptor preference for the F2Pal_16_ pepducin include neutrophils isolated from Fpr2 deficient (Fpr2^-/-^) mice and the recently described selective ligands for mouse Fprs [12, 13]. The F2Pal_16_-induced response was significantly lower in neutrophils isolated from Fpr2^-/-^ mice compared to that from WT cells (Fig 2A), suggesting that Fpr2 is the receptor for F2Pal_16_. This suggestion gained further support from receptor desensitization experiments using the Fpr2 selective agonist PSMα2 [12]. Cells activated with PSMα2 were non-responding (desensitized) to a second stimulation with F2Pal_16_ (Fig 2B). The same desensitization profile was obtained when the order of agonist addition was reversed (Fig 2B). In contrast, no such desensitization was obtained when PSMα2 was replaced with the Fpr1 specific agonist fMIFL (Fig 2B inset). Further, the F2Pal_16_ response in WT cells was significantly reduced by the earlier described Fpr2 selective peptidomimetic inhibitor (Lau-(Lys-βNSpe)_6_-NH_2_) but not the Fpr1 antagonist Boc2 (Fig 2C). The fact that the remaining F2Pal_16_ response in Fpr2^-/-^ cells was insensitive to Boc2 indicates that Fpr1 is not targeted by F2Pal_16_ (Fig 2D). For comparison, the response induced by the very potent Fpr1 agonist fMIFL was completely abolished by Boc2 (Fig 2D). In summary, these data show that the F2Pal_16_ pepducin generated from the third intracellular loop of FPR2 positively modulate not only FPR2 but also its mouse orthologue Fpr2.

### The inverse relationship between peptide length and FPR2 activation potency does not apply for Fpr2

**Fig 3:**
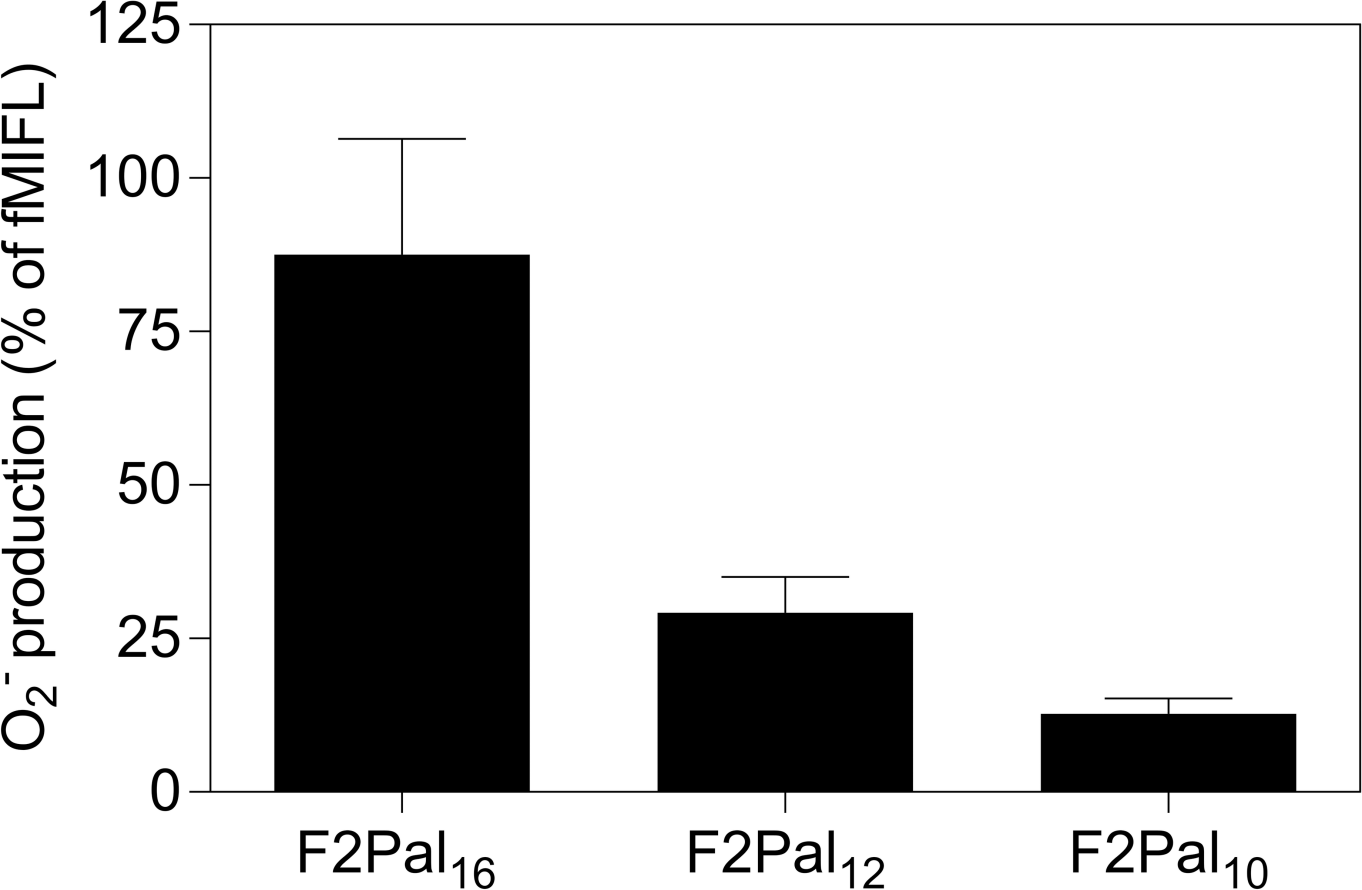
The superoxide release induced by the shorter F2Pal_16_ derivatives in mouse neutrophils. WT mouse neutrophils (5x10^4^ cells) were activated with 1 μM of F2Pal_16_ and its C-terminal truncated derivatives F2Pal_12_ and F2Pal_10_ after which the release of superoxide anions was continuously recorded. The relative activity of pepducins is expressed in percent of the peak value obtained by the fMIFL response (10 nM) (mean ± SD) from three independent experiments with individual mouse.

We have earlier shown that the FPR2 activation potency of F2Pal_16_ is affected both by the peptide and the N-terminally conjugated fatty acid [20, 32]. The fatty acid was found to be required for activity and there was an inverse relationship between activity and the length of the peptide chain with F2Pal_10_ being the most potent agonist for FPR2. The impact of the fatty acid and the length of the peptide chain were evaluated in mouse neutrophils and we found that the fatty acid was needed also for activation of mouse neutrophils, but, in contrast to human neutrophils, F2Pal_16_ was more potent than shorter derivatives (Fig 3).

### The mF2Pal_16_ pepducin generated from the third intracellular loop of Fpr2 negatively modulates both Fpr2 and FPR2

**Fig 4:**
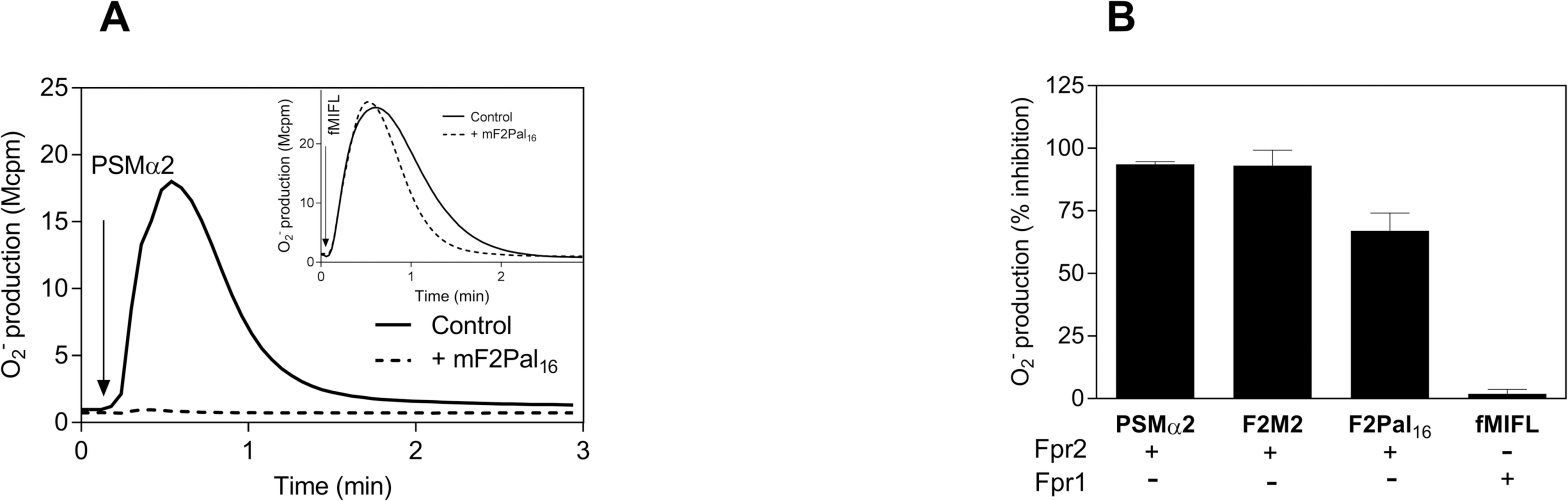
The mF2Pal_16_ pepducin generated from Fpr2 inhibits the activity of Fpr2 in mouse neutrophils. **A)** WT mouse neutrophils (5 x 10^4^ cells) were pre-incubated without (solid line) or with mF2Pal_16_ (500 nM) for 5 min before stimulation with the peptide PSMα2 (10 nM, indicated by the arrow) and the release of superoxide anions was continuously measured. **Inset:** The fMIFL response (10 nM) in the presence (dashed line) or absence (solid line) of mF2Pal_16_ (1 μM). **B)** The percent of inhibition on the response induced by PSMα2 (10 nM), F2M2 (150 nM), F2Pal_16_ (500 nM) and fMIFL (10 nM) was calculated from the each individual agonist peak response in the absence of mF2Pal_16_ (500 nM, mean ± SD) from three independent experiments with individual mouse.

The results obtained with the FPR2 derived pepducin F2Pal_16_ clearly show that although the amino acids in the third intracellular loop FPR2 differ in seven positions from those in Fpr2, the F2Pal_16_ pepducin targeted Fpr2 but not Fpr1, a receptor differing in only three positions from the F2Pal_16_ sequence (Table 1). This prompted us to challenge the pepducin concept and determine the activities induced by the Fpr2 pepducin (mF2Pal_16_, Pal-KINRRNLVNSSRPLR). No activation of mouse neutrophils was obtained using mF2Pal_16_, however, since pepducins may negatively modulate the targeted receptor, we examined the effects also in "inhibitory mode experiments". Our data show that mF2Pal_16_ potently inhibited the response induced by the Fpr2 specific agonist PSMα2 with an IC_50_ ~ 30 nM (Fig 4A) but there was no effect on the response induced by the Fpr1 agonist fMIFL (Fig 4B). The inhibitory effect of mF2Pal_16_ pepducin was observed also when PSMα2 was replaced by two other Fpr2 selective agonists, i.e., the peptidomimetic agonist F2M2 and F2Pal_16_ (Fig 4B). Taken together, these data show that mF2Pal_16_ is an Fpr2 selective inhibitor.

**Fig 5:**
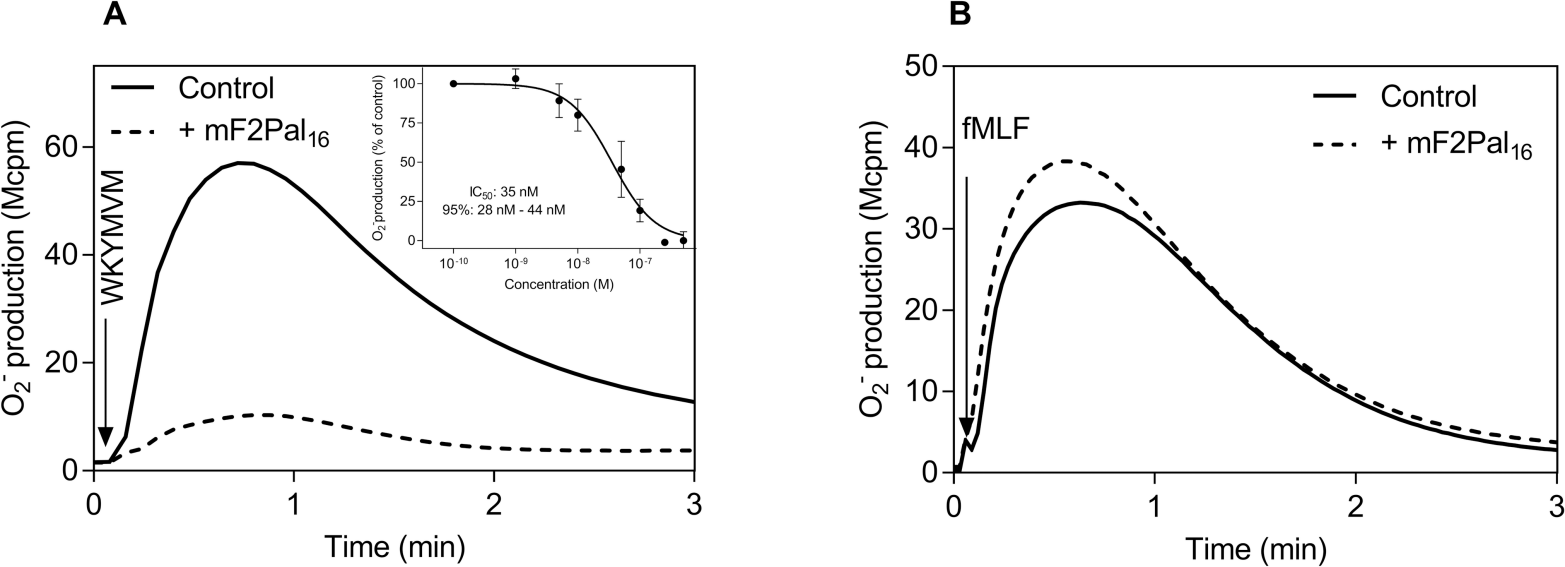
The mF2Pal_16_ pepducin inhibits the activity of FPR2 in human neutrophils. Human neutrophils (10^5^ cells) were pre-incubated without (solid line) or with mF2Pal_16_ (1 μM) for 5 min before stimulation with **A**) the FPR2 specific agonist WKYMVM (50 nM, indicated by the arrow). **Inset:** Dose-dependent activation of mouse neutrophils by mF2Pal_16_. Data are normalized to the maximal response and curve fillting by non-linear regression using the sigmoidal dose-response equation (variable-slope, HillSlope of -1.3) was used. Curve fitting was performed by **B**) effect of mF2Pal_16_ (1 μM) on the FPR1 specific agonist fMLF (50 nM, indicated by the arrow). The release of superoxide anions was measured continuously. Representative responses out of five experiments from individual buffy coats are shown.

In agreement with the results obtained in mouse neutrophils, mF2Pal_16_ did not activate human neutrophils to produce superoxide, but the responses induced by the FPR2 specific agonists WKYMVM and PSMα2 were largely inhibited (Fig 5A). The inhibitory effect of mF2Pal_16_ was not of the same magnitude as that of the most potent FPR2 inhibitor PBP_10_, but still the inhibitory effect obtained was receptor specific as illustrated by the lack of inhibitory effect induced by the FPR1 specific agonist fMLF (Fig 5B). Our data, thus, disclose mF2Pal_16_ as a potent and selective inhibitor for Fpr2/FPR2 and F2Pal_16_ as a selective activator of Fpr2/FPR2.

### The FPR2 inhibitory pepducin F1Pal_16_ also modulates mouse neutrophil functions but the outcome is switched from inhibition into activation

**Fig 6:**
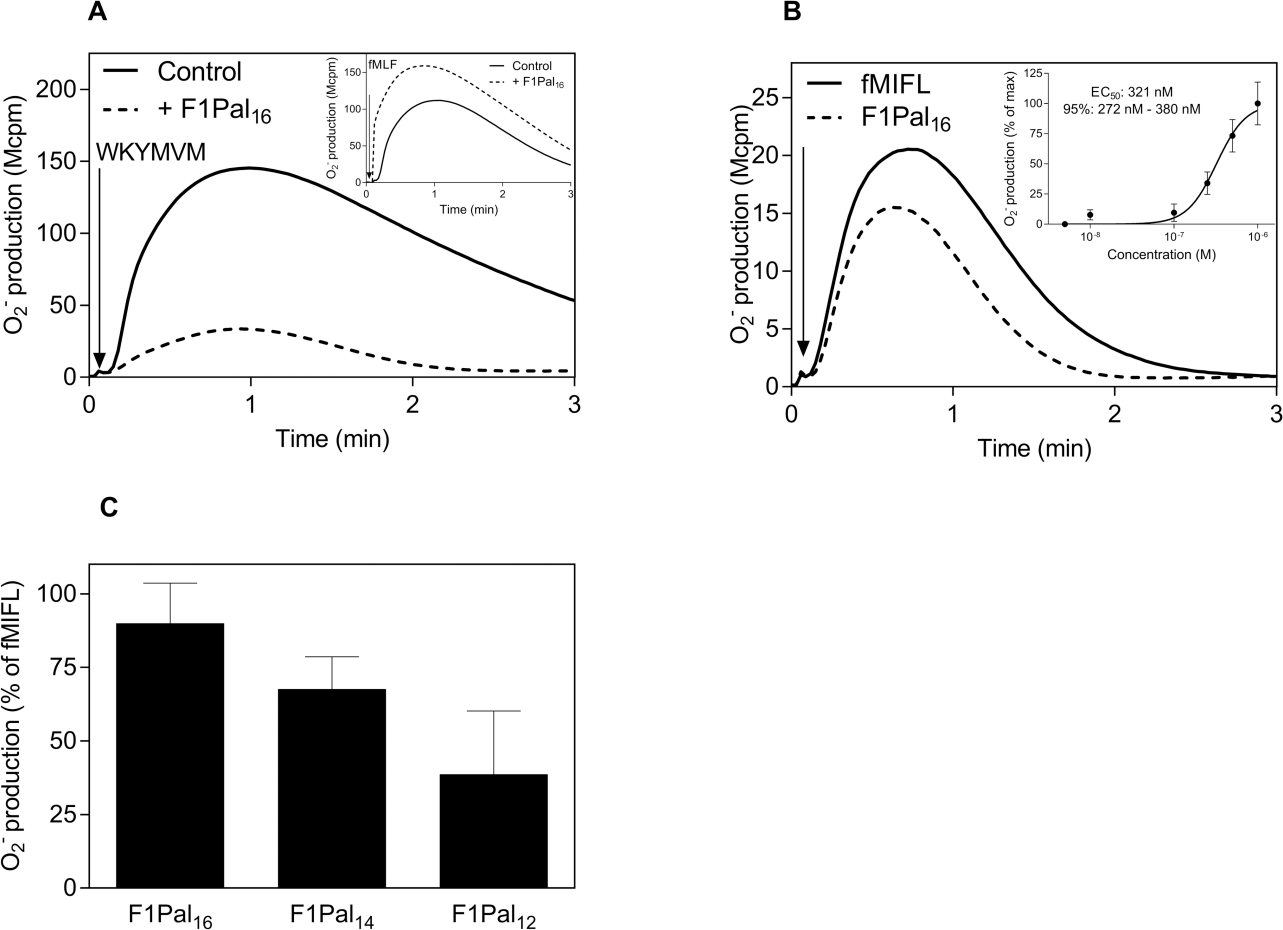
The F1Pal_16_ pepducin generated from FPR1 inhibits the activity of FPR2 in human neutrophils but activates mouse neutrophils. **A)** Human neutrophils (10^5^ cells) were pre-incubated with F1Pal_16_ (1 μM, dashed line) or without (solid line) for 5 min before stimulation with the FPR2 agonist WKYMVM (50 nM, indicated by the arrow). The release of superoxide anions was continuously measured. The figure shows one representative experiment out of five independent experiments from individual buffy coats. **Inset**: Cells were pre-incubated with F1Pal_16_ (1 μM, dashed line) for 5 min before stimulation with the FPR1 agonist, fMLF (50 nM, indicated by the arrow, dashed line) or left untreated (solid line). **B)** Mouse neutrophils (5 x 10^4^ cells) were activated by addition of the pepducin F1Pal_16_ (1 μM, indicated by the arrow). The figure shows one representative experiment out of five. **Inset:** Dose-dependent activation of WT mouse neutrophils by F1Pal_16_. Data are normalized to the maximal response and curve fitting was performed by non-linear regression using the sigmoidal dose-response equation (variable-slope, HillSlope 2.5). EC_50_ value and 95% confidence interval (n = 3, mean ± SD). **C)** Mouse neutrophils (5x10^4^ cells) were activated with F1Pal_16_ (1 μM) and the C-terminal truncated peptides F1Pal_14_ and F1Pal_12_, and the release of superoxide anions was continuously recorded. The relative activity of pepducins is expressed in percent of the peak value obtained by the fMIFL (10 nM) response (mean ± SD) from three independent experiments with individual mouse.

The FPR1 pepducin F1Pal_16_ (Pal-KIHKQGLIKSSRPLRV) interacts with FPR2 and inhibits this receptor specifically [23]. We now confirm that F1Pal_16_ inhibit the response induced by the FPR2 agonist WKYMVM (Fig 6A) but not the FPR1 agonist fMLF (Fig 6A inset). In direct opposition to the negative modulating effects observed in human neutrophils, F1Pal_16_ positively modulated mouse neutrophils and directly triggered the release of superoxide with an activation kinetics resembling that of fMIFL (Fig 6B). Mouse neutrophil activation by F1Pal_16_ was concentration dependent with an EC_50_ value ~ 300 nM (Fig 6B inset), which is comparable to that of the FPR2 derived pepducin F2Pal_16_ (Fig 1B). In addition, the response was sensitive to the pepducin inhibitor mF2pal_16_ identified above in this study (S1 Fig).

We have earlier shown that the full length pepducin F1Pal_16_ is a potent FPR2 inhibitor [23]. In this study, we show that F1Pal_16_ with 16-mer peptide was also the most potent in activating mouse neutrophils among its derivatives with shorter peptide chains (Fig 6C). The last four amino acids from the C-terminus in F1Pal_16_ have thus the same impacts on neutrophil modulation across species. For the F2Pal-pepducins, there is an inverse relationship between peptide length in F2Pal and activation potency for FPR2 but not for Fpr2 (Fig 3).

### The FPR1/Fpr1 pepducins inhibit FPR2 function and activate its mouse orthologue Fpr2

**Fig 7:**
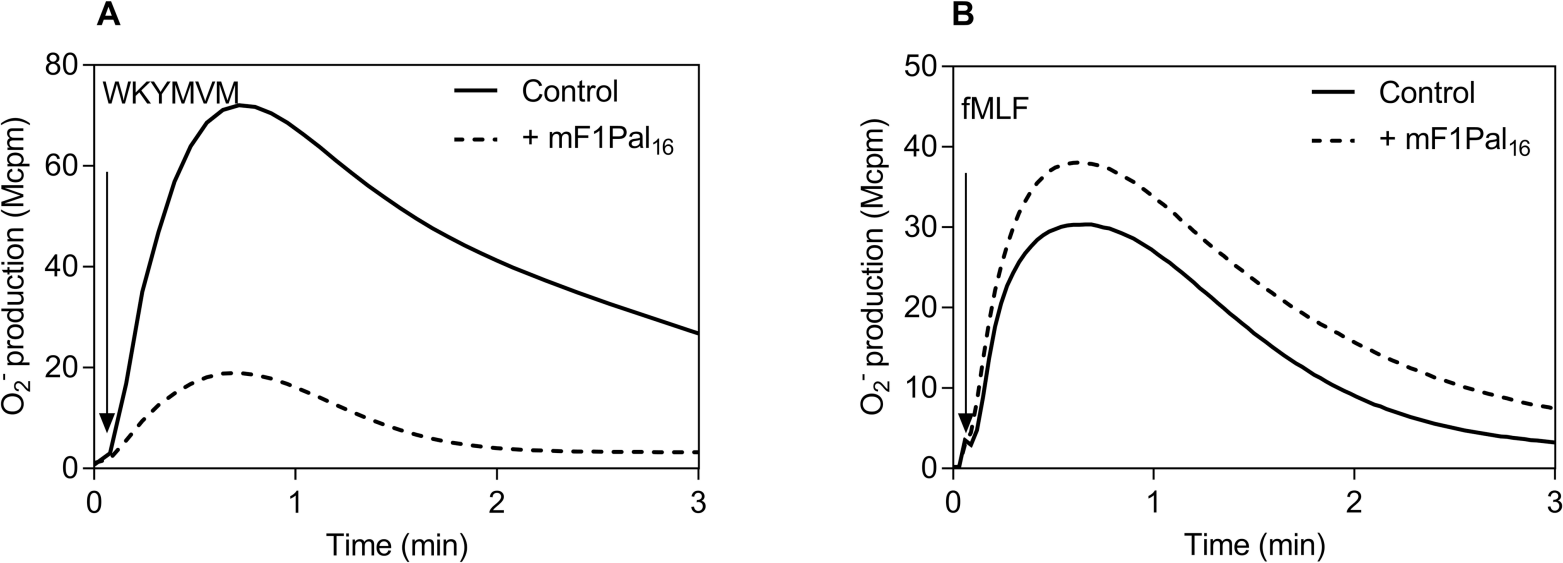
The mF1Pal_16_ pepducin generated from Fpr1 inhibits the FPR2 activity in human neutrophils. **A)** Human neutrophils (10^5^ cells) were pre-incubated without (solid line) or with mF1Pal_16_ (1 μM, dashed line) before stimulation with FPR2 agonist WKYMVM (50 nM, indicated by the arrow) and the release of superoxide anions was continuously measured. The figure shows one representative experiment out of four independent experiments with individual buffy coats. **B)** Human neutrophils (10^5^ cells) were pre-incubated without (solid line) or with mF1Pal_16_ (1 μM, dashed line) before stimulation with the FPR1 agonist fMLF (50 nM, indicated by the arrow) and the release of superoxide anions was continuously measured. The figure shows one representative experiment out of four independent experiments with individual buffy coats.

FPR1 shares 72% sequence identity at the amino acid level to its mouse orthologue Fpr1, and they differ in only one amino acid in the third intracellular loops from which the Fpr1 pepducin mF1Pal_16_ and FPR1 pepducin F1Pal_16_ are generated (Table 1). This amino acid (K in F1Pal_16_ instead of R in mF1Pal_16_ at the fourth position) is obviously not critical for pepducin action as the two pepducins induced very similar activities but with somewhat different potency. Human neutrophils were not activated by mF1Pal_16_ (tested in concentrations up to 1 μM; S2 Fig); however, mF1Pal_16_ inhibited the response induced by the FPR2 specific agonists WKYMVM (Fig 7A). Similar to F1Pal_16_, mF1Pal_16_ displayed preference for FPR2 over FPR1 as it did not affect the response induced by the FPR1 specific agonist fMLF (Fig 7B).

**Fig 8:**
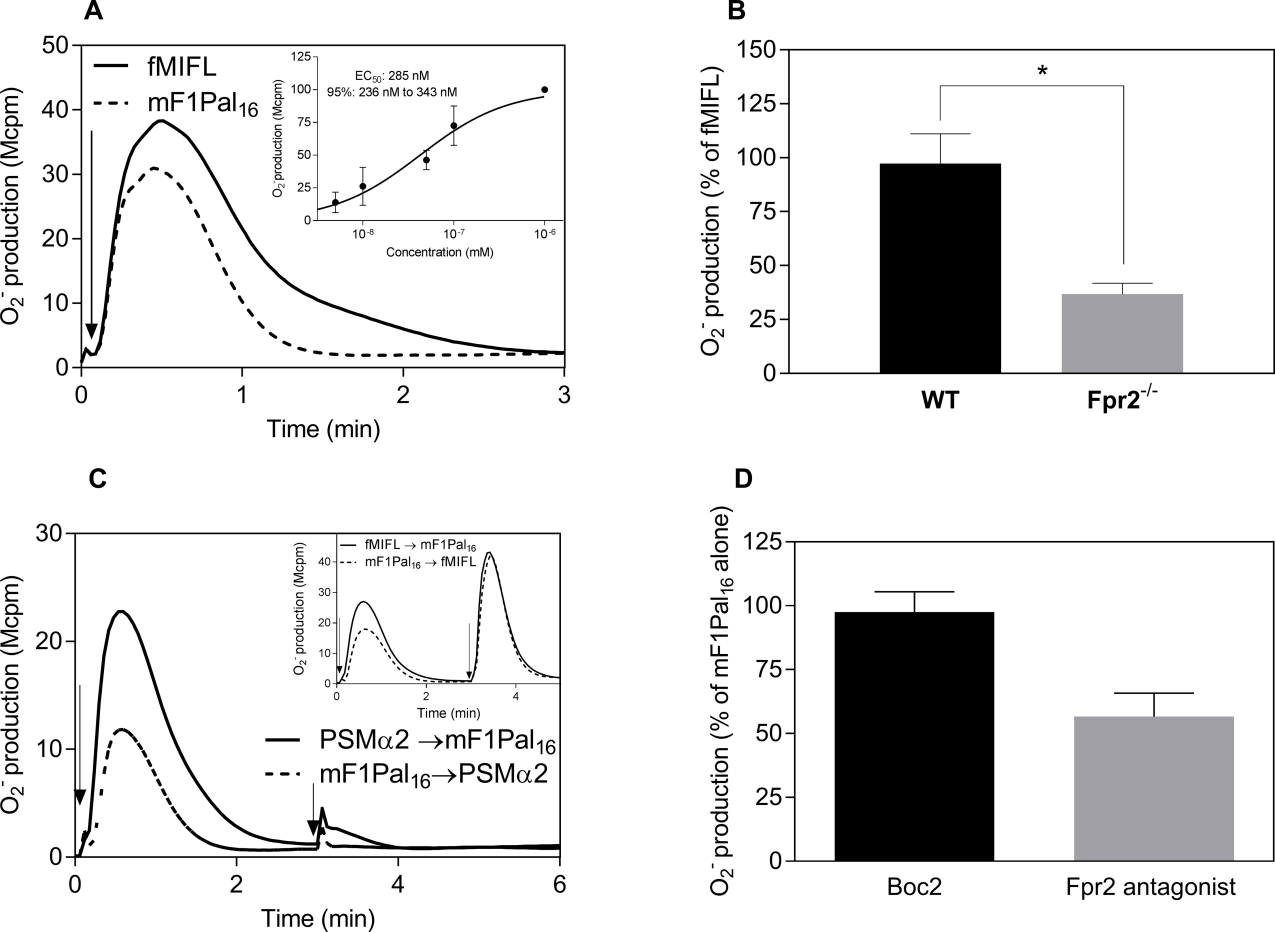
The mF1Pal_16_ pepducin activates primarily Fpr2 in mouse neutrophils. **A)** Mouse neutrophils (5 x 10^4^ cells) were activated by addition of the mF1Pal_16_ pepducin (1 μM). **Inset:** Dose-dependent activation of mouse neutrophils by mF1Pal_16_. Data is normalized to the maximal response and curve fitting was performed by non-linear regression using the sigmoidal dose-response equation (variable slope, HillSlope 0.9). EC_50_ value and 95% confidence interval (mean ± SD) were from three independent experiments with individual mouse. **B)** The mF1Pal_16_ induced cell activation in mouse neutrophils (5 x 10^4^ cells) isolated from WT (black bar) and Fpr2^-/-^ (grey bar) mice. Cells were incubated for 5 min at 37°C before the addition of mF1Pal_16_ (1 μM) and the NADPH-oxidase activity was measured over time. The peak values obtained were compared and the results are expressed as percent of the peak activity induced by fMIFL (10 nM) in cells derived from each individual mouse (mean ± SD) from three independent experiments with individual mouse. * *p* ≤ 0.05. **C)** Cross-desensitization between PSMα2 (50 nM) and mF1Pal_16_ (1 μM) in WT mouse neutrophils. Cells first received (indicated by the first arrow) PSMα2 (solid line) or mF1Pal_16_ (dashed line) and when the response had declined, a second stimulation (indicated by the second arrow) was induced by mF1Pal_16_ (solid line) or PSMα2 (dashed line). **Inset:** Cross-desensitization between mF1Pal_16_ and fMIFL in mouse neutrophils. Cells first received (indicated by the first arrow) fMIFL (10 nM, solid line) or mF1Pal_16_ (1 μM, dashed line) and when the response had declined, a second stimulation (indicated by the second arrow) was induced by mF1Pal_16_ (1 μM, solid line) or fMIFL (10 nM, dashed line). The release of superoxide anions was continuously measured. Representative curves out of three independent experiments from individual mouse are shown. **D)** Effect of Fpr1 inhibitor Boc2 (2 μM, black bar) and Fpr2 antagonist (1 μM, grey bar) on the mF1Pal_16_ (500 nM) response. The data are from five independent experiments with individual mouse (mean ± SD) and One-way ANOVA with Dunnett’s multiple comparisons test in comparison to the mF1Pal_16_ control response was used for statistics.

The mF1Pal_16_ pepducin triggered activation of the NADPH-oxidase in mouse neutrophils and the activity was concentration dependent with a much lower EC_50_ value as compared to that of F1Pal_16_ (Fig 8A and Fig 6B). Similar to F2Pal_16_, the response induced by mF1Pal_16_ was not abolished but significantly reduced in Fpr2^-/-^ cells (Fig 8B). Receptor desensitization patterns showed that PSMα2-stimulated, but not fMIFL-stimulated cells were non-responsive to a second stimulation with mF1Pal_16_ in mouse neutrophils (Fig 8C). In addition, the Fpr2 peptidomimetic inhibitor (Lau-(Lys-βNSpe)_6_-NH_2_) but not the Fpr1 inhibitor Boc2 (Fig 8D). Very similar data were obtained from Fpr2^-/-^ and receptor inhibition and desensitization experiments when mF1pal_16_ was replaced by F1Pal_16_. Taken together, we show that FPR1/Fpr1 pepducins utilize FPR2 in human and the orthologue Fpr2 in mouse, but with different modulating outcomes (negative on FPR2 and positive on Fpr2).

## Discussion

The formyl peptide receptors play important roles in host defense and as immune regulator [1, 2], suggesting functional receptor modulation could be the basis for the development of FPR-based therapeutics. N-terminal lipidated peptides (pepducins) constitute a novel class of allosteric functional modulators of GPCRs supposedly acting at the signaling interface of the targeted receptor [15–17]. We show in this study that pepducins derived from the third intracellular loops of formyl peptide receptors (FPR1/FPR2 in human and Fpr1/Fpr2 in mouse) either activate or inhibit neutrophils. The active pepducins all affected the function of one of the family members (FPR2 in human and Fpr2 in mouse), without influencing the closely related FPR1/Fpr1. Although pepducins are excellent tool-molecules in fine-tuning the basic functions of FPR/Fpr, our data bring up some important issues related to the use of mouse models for FPR studies and to the pepducin concept/mechanism of action, that need to be critically considered and further investigated.

Pepducins with a peptide sequence identical to one of the intracellular domains or cytoplasmic tail of a GPCR were introduced in the early 21^th^ century as a novel concept for allosteric modulation of GPCR signaling [15, 16, 33]. The mechanism suggested for how the allosteric modulation and receptor selectivity was achieved, turned the two-state receptor model predominant at that time upside-down, or rather turned it outside-in [34]. The suggested mode of action for pepducins states that the fatty acid, due to its physico-chemical properties, anchors the pepducin to the plasma membrane and the peptide part of the molecule is then flipped across plasma membrane where it interferes with the cytosolic signaling parts of the receptor [16, 18]. According to the model, the peptide part of a pepducin determines the receptor specificity that is dependent by the amino acid identity between the pepducin and the targeted receptor from which pepducin sequence originates. Even if it is hard to understand the basic mechanism for how the peptide part of a pepducin translocates to the inner leaflet of the membrane and how two identical peptide sequences interact and by that either inhibits or activates receptor signaling, the model creates some in-built restrictions, i) a pepducin should only activate/inhibit the function of receptors that contains an identical sequence in one of the intracellular signaling domains, and ii) conventional antagonists that block the orthosteric binding site of the receptor at the extracellular surface should not affect the activity induced by a receptor activating pepducin. The data presented in this study together with our earlier studies are not always consistent with these restrictions [24, 25]. Several FPR2 activating pepducins are sensitive to conventional FPR2 antagonists and likewise, binding experiments reveal that conventional agonists compete with a pepducin for receptor binding [20]. Nevertheless, all pepducins described to date require the presence of a fatty acid to mediate receptor modulation. The fatty acid alone is, however, not sufficient for receptor modulation and membrane association does not necessarily induce membrane translocation of the conjugated peptide. Even if some peptides have the capacity to penetrate the cell membrane barrier, the biological response induced is not necessarily the consequence of an interaction with the receptor from the cytosolic side of the membrane but can be evoked by an interaction with allosteric/orthosteric binding sites present in/on surface exposed parts of the receptor. It is important to also point out that for all pepducins described (including those that target FPR2) the amino acid sequence in the peptide part is of prime importance for receptor interaction, and in accordance with this there are many palmitic acid conjugated peptides not recognized by FPR2 [25, 35].

In agreement with the pepducin concept regarding origin and receptor specificity, the pepducin derived from the third intracellular loop of FPR2 targets FPR2, and the one derived from Fpr2 (mF2Pal_16_) targets this receptor ([19, 20], this study). The FPR/Fpr pepducins are receptor-selective although the functional link (activating or inhibiting) between the targeted receptor and the peptide sequences of the pepducins is missing. In contrast to FPR2/Fpr2 pepducins, FPR1- and Fpr1-derived pepducins designed from their respective third intracellular loops lack FPR1/Fpr1 modulating effects ([24], this study). The close sequence similarities between the third intracellular loops in FPR1 and FPR2 (Table 1) could possibly explain why the FPR1 pepducin targets also FPR2. This reasoning can, however, not be used to explain the lack of effects of this pepducin on FPR1 or the cross-reactivity of the FPR1/FPR2 pepducins on Fpr2, a receptor that differs substantially from FPR1/FPR2 in the third intracellular loop (Table 1). Our data obtained with the two FPR1/Fpr1 pepducins (F1Pal_16_ and mF1Pal_16_) indicates that there is no fundamental difference in signaling profile between human and mouse derived peptides, as these two pepducins are almost identical (one amino acid difference) and they have the same effects, i.e., they negatively target human FPR2 and positively target the mouse Fpr2. This suggests that their effects can only be explained by the differences between FPR2 and Fpr2 across species. More importantly, they don’t target the receptor FPR1/Fpr1 predicted by the pepducin specificity concept. Instead, our data strongly suggests that some specific motif in FPR2/Fpr2 may recognize a lipopeptide pattern, as suggested by the fact that this receptor seems to be the primary target for pepducins derived also from other, sometimes totally unrelated, GPCRs [24, 25]. This suggestion gains further support from the identification of a novel class of lipidated peptoids (peptidomimetics) as FPR2/Fpr2 interacting ligands [12, 36, 37]. It is worth noting that although the lipid part is essential, it is not alone sufficient to interact with FPR2. As mentioned there are many fatty acid conjugated peptides that are not recognized by FPR2 [24, 25, 38]. The precise binding site for pepducins has not been worked out, but our earlier data show that an FPR2 pepducin activates also an FPR chimera in which the precise pepducin sequence is missing [20]. Further, data generated with chimeric FPRs has demonstrated that a replacement of the cytoplasmic tail (not involved in binding of any ligand) of FPR1 with that from FPR2 abolishes the ability of this receptor to recognize FPR1 ligands, whereas the receptor gains affinity for FPR2 ligands including the pepducins [39]. Formation of a high-affinity agonist binding site in FPRs is, thus, a highly dynamic process that involves a coordinate folding of multiple domains including sites that are not directly involved in binding. Taken together, a number of different questions remain to be answered including the precise mechanism of action of pepducins and the identity of a common molecular motif (if any) present in FPR2/Fpr2 interacting pepducins, and future studies could possible identify natural inflammatory mediators (of microbial or endogenous origin) that contain a molecular pattern recognized by the innate immune system through FPR2/Fpr2.

All activating pepducins examined in this study target not only FPR2 in human neutrophils but also the mouse neutrophil Fpr2. The C-terminus in these pepducins contain 6 amino acids that are identical in all of them, and our data from this and earlier published studies show that this part is of prime importance for activity. This is illustrated by the fact that upon a removal of this part from F1Pal_16_ results in a reduction of the inhibitory effect on FPR2 as well as the activating effect on Fpr2 ([23], this study). When removed from F2Pal_16_ the activation potency is increased on FPR2 but decreased on Fpr2 ([20], this study). It is clear that the activity induced by a pepducin depends not only on the amino acid sequence and the length of the peptide but also on its charge and the length of fatty acid. With respect to FPR2 pepducin we have shown that the fifth amino acid (the K_5_ residue) in the FPR2 pepducins is of prime importance for FPR2 activation; the K_5_ residue could be replaced by an R (present in mF2Pal_16_) without any activity major change, but when replaced by Q (present in F1Pal_16_ and in mF1Pal_16_) the agonistic effect is completely lost [20]. In addition, structure-activity relationship studies with the F2Pal pepducin-derivatives show that the position of the charged amino acids rather than the net charge of the peptide determines the FPR2 agonistic activity of the pepducin [22], and this is in line with the data derived from FPR2 interacting peptidomimetics [35]. Our data from this study show that the activating pepducins also activate neutrophils isolated from Fpr2 deficient animals, suggesting that yet another receptor is involved. The fact that the Fpr1 specific inhibitor Boc2 [12] has no effect on the pepducin response either in WT or Fpr2^-/-^ cells strongly suggests that Fpr1 is not targeted by these pepducins. Although according to the pepducin concept an extracellular receptor antagonist, including Boc2, should not affect the pepducin activity, our earlier studies have demonstrated that this rule does not apply to FPR2 activating pepducins [20]. The human genome encodes three members of the FPR family (FPR1-FPR3). FPR2 shares 72% homology with FPR3 that is not expressed by neutrophils but by monocytes, and many FPR2 ligands cross-react with FPR3 [2]. Thus, the mouse FPR3 orthologue could be the receptor candidate for these pepducins. However, the promiscuous binding profile of FPRs and their complex evolutionary history across species make it difficult to accurately define the orthologship of these genes in human and mouse. In addition, FPR3 selective agonists or antagonists are still lacking, making it difficult to target FPR3 without cross-interacting with FPR2. With respect to this, we have attempted to apply the pepducin approach to target FPR3, but without success. Future identification of tools that selectively target FPR3/Fpr3 would help delineate the role of this receptor member in the response to pepducins both in human and in mouse.

Genetically modified mouse strains and mouse disease models provide excellent tools to study the function of neutrophil FPRs *in vivo*. Such studies have indicated Fprs (in particular Fpr2) as important regulators of the physiological processes leading to resolution of inflammation [1]. However, a direct translation of mouse data to human studies is hampered due to the distant phylogenetic relationship between mouse and human receptors [40]. In addition to the different number of *Fpr* genes in mice and humans and an incomplete understanding of the evolutionary relationship between human FPRs and their animal counterparts, also the fine-tuning of receptor ligands has obvious implications for how the results obtained should be interpreted [12]. A large number of agonists for the FPRs expressed in human neutrophils isolated from peripheral blood have been identified and characterized for their precise receptor preference using a fairly limited number of available antagonists/inhibitors. It is intriguing that the potent FPR1-specific agonist fMLF as well as the potent FPR2 agonist WKYMVM display low affinity for their mouse orthologous, and that some agonists and antagonists that potently affect FPRs lack effects in relation to Fprs [12]. We now add to this that an FPR2 antagonist (F1Pal_16_ or mF1Pal_16_) can act as an agonist for Fpr2, and our data demonstrate new agonistic and antagonistic pepducins that might be useful tools for future translational studies in mouse models. These two FPR1/Fpr1-derived pepducins are almost identical in sequence (one amino acid difference) and exert similar effects, i.e., they both negatively target human FPR2 and positively target the mouse Fpr2. This suggests that there is no pharmacological profile difference between the two pepducins and the difference must be due to differences between the human and mouse receptors. More importantly, our data puts the pepducin specificity concept into question as these two pepducins don’t target FPR1/Fpr1 as expected. Availability of these well-characterized cross-species ligands is important to advance mouse disease models aimed at understanding the role of human FPRs in immune defense and inflammation. Our data reveal clearly that even small structural variations may abolish cross-species ligand recognition and this holds true for both agonists and antagonists. Cross-species receptor ligands for members of the FPR family, possessing a high degree of receptor-selectivity, are required for translation of activation/inhibition experiments performed with different animal disease models. Using mouse models for innate immune research always raises questions about species-specific differences that have to be considered when extrapolating results from the models to human diseases, but also there are reports describing differences in neutrophil biology between species. For example, mouse neutrophils do not produce defensins, an important group of microbial peptides produced by human neutrophils [40].

In conclusion, we have identified both activating and inhibiting pepducins for mouse neutrophils and these pepducins display a receptor preference for Fpr2. Our data also highlight differences in ligand recognition between FPR2 and Fpr2 that have to be taken into account when choosing receptor specific ligands for translational studies across species. A better understanding of Fpr2 pharmacology is needed and will facilitate *in vivo* studies related to the pathophysiologic functions of FPR2. With its complexity in ligand binding and signaling modulation FPR2 provides an excellent model receptor for our understanding of GPCR signaling and modulation in general, and the potent and unique FPR2/Fpr2 modulating pepducins presented in this study could be excellent tools in future mechanistic and functional studies.

## Supporting information

S1 Fig The mF2Pal_16_ pepducin generated from Fpr2 inhibits the activity of F1Pal_16_ in mouse neutrophils. WT mouse neutrophils (5 x 10^4^ cells) were pre-incubated without (solid line) or with mF2Pal_16_ (250 nM) for 5 min before stimulation with the pepducin F1Pal_16_ (500 nM, indicated by the arrow) and the release of superoxide anions was continuously measured. One representative experiment out of three performed with individual buffy coats is shown.
(TIF)

S2 Fig The mF1Pal_16_ pepducin generated from FPR1 does not activate human neutrophils to produce superoxide. Human neutrophils (10^5^ cells) were pre-incubated with HRP and isoluminol followed by stimulation with the FPR2 specific agonist WKYMVM (100 nM, solid line) or the pepducin mF1Pal_16_ (1 μM, dotted line). Arrow indicates the addition. The release of superoxide anions was continuously measured. One representative experiment out of five independent experiments performed with individual buffy coats is shown.
(TIF)
