# Supplementary figures and images for: Formyl peptide derived lipopeptides disclose differences between the receptors in mouse and men and call the pepducin concept in question
Source: PLoS One. 2017 Sep 21;12(9):e0185132. doi: 10.1371/journal.pone.0185132 (PMC5608352; doi:10.1371/journal.pone.0185132)

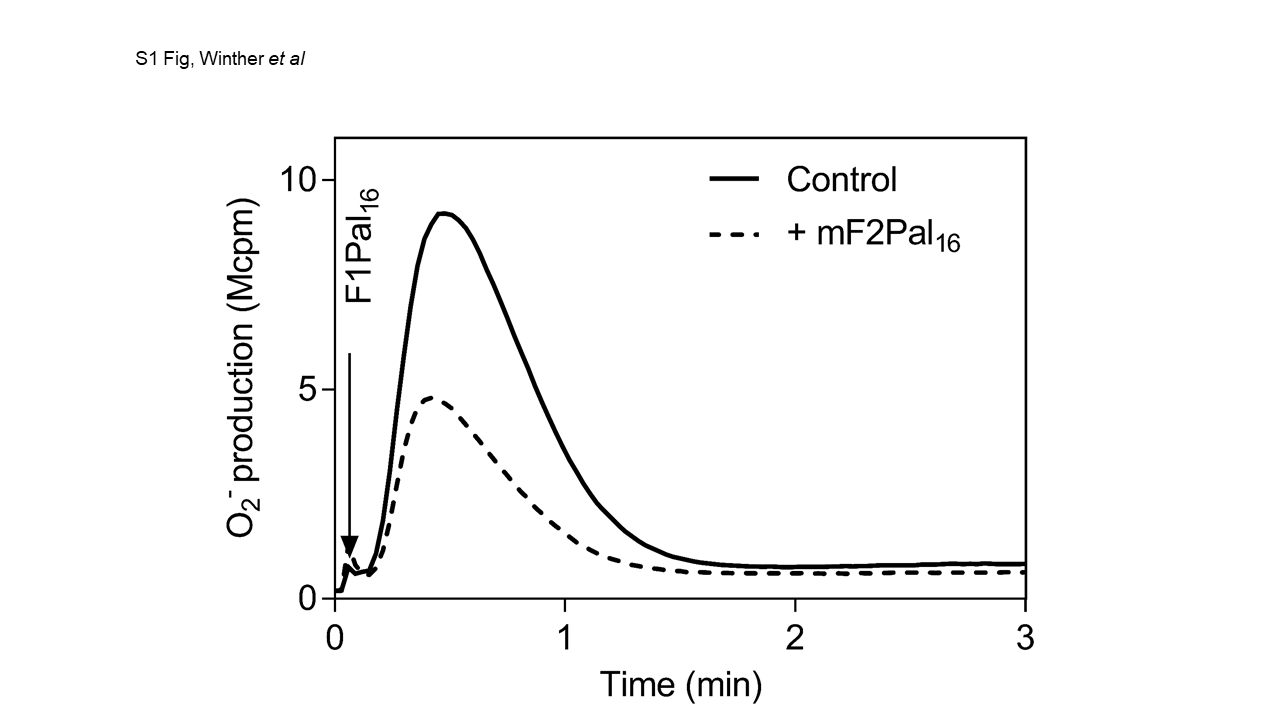

Supplement: S1 Fig — WT mouse neutrophils (5 x 10^4^ cells) were pre-incubated without (solid line) or with mF2Pal16 (250 nM) for 5 min before stimulation with the pepducin F1Pal16 (500 nM, indicated by the arrow) and the release of superoxide anions was continuously measured. One representative experiment out of three performed with individual buffy coats is shown. (TIF) [file pone.0185132.s001.tif]

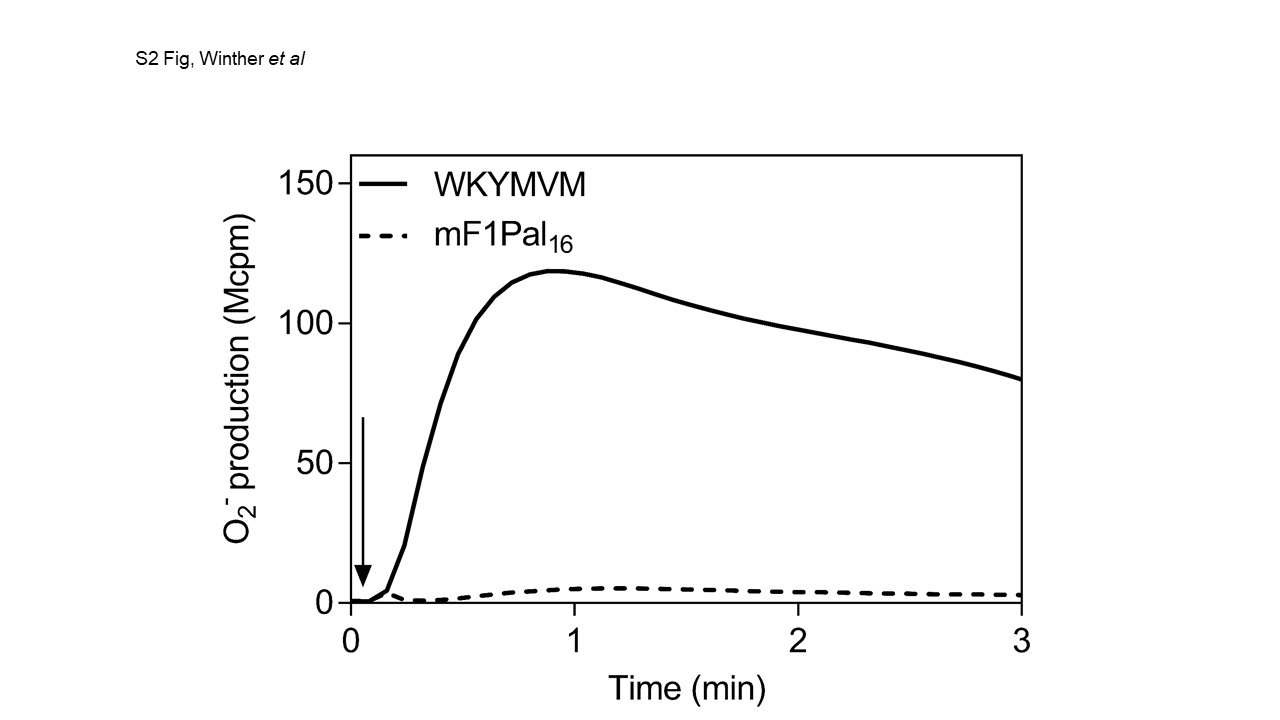

Supplement: S2 Fig — Human neutrophils (105 cells) were pre-incubated with HRP and isoluminol followed by stimulation with the FPR2 specific agonist WKYMVM (100 nM, solid line) or the pepducin mF1Pal16 (1 μM, dotted line). Arrow indicates the addition. The release of superoxide anions was continuously measured. One representative experiment out of five independent experiments performed with individual buffy coats is shown. (TIF) [file pone.0185132.s002.tif]
